# Independent evolution of intermediate bill widths in a seabird clade

**DOI:** 10.1007/s00438-021-01845-3

**Published:** 2021-12-18

**Authors:** Juan F. Masello, Peter G. Ryan, Lara D. Shepherd, Petra Quillfeldt, Yves Cherel, Alan J. D. Tennyson, Rachael Alderman, Luciano Calderón, Theresa L. Cole, Richard J. Cuthbert, Ben J. Dilley, Melanie Massaro, Colin M. Miskelly, Joan Navarro, Richard A. Phillips, Henri Weimerskirch, Yoshan Moodley

**Affiliations:** 1 Department of Animal Ecology and Systematics, Justus Liebig University Giessen, Heinrich-Buff-Ring 26, 35392 Giessen, Germany; 2 FitzPatrick Institute of African Ornithology, DST-NRF Centre of Excellence, University of Cape Town, Rondebosch, 7701 South Africa; 3 Museum of New Zealand Te Papa Tongarewa, PO Box 467, Wellington, 6140 New Zealand; 4 Centre d’Etudes Biologiques de Chizé, UMR 7372 CNRS, La Rochelle Université, 79360 Villiers-en-Bois, France; 5 Department of Primary Industries, Parks, Water and Environment, GPO Box 44, Hobart, TAS 7001 Australia; 6 Instituto de Biología Agrícola de Mendoza (IBAM, CONICET-UNCuyo), Almirante Brown 500, M5528AHB Chacras de Coria, Mendoza Argentina; 7 Department of Biology, Ecology and Evolution, University of Copenhagen, Universitetsparken 15, 2100 Copenhagen, Denmark; 8 Royal Society for the Protection of Birds, The Lodge, Sandy, SG19 2DL UK; 9 School of Environmental Sciences and Institute for Land, Water and Society, Charles Sturt University, PO Box 789, Albury, NSW 2640 Australia; 10 Institut de Ciències del Mar ICM-CSIC, Passeig Maritim de la Barceloneta 37-49, 08003 Barcelona, Spain; 11 British Antarctic Survey, Natural Environment Research Council, High Cross, Madingley Road, Cambridge, CB3 0ET UK; 12 Department of Zoology, University of Venda, Private Bag X5050, Thohoyandou, 0950 South Africa

**Keywords:** Convergent evolution, Gough Island, MacGillivray’s prion, *Pachyptila*, Procellariidae, Procellariiformes

## Abstract

Interspecific introgression can occur between species that evolve rapidly within an adaptive radiation. *Pachyptila* petrels differ in bill size and are characterised by incomplete reproductive isolation, leading to interspecific gene flow. Salvin’s prion (*Pachyptila salvini*), whose bill width is intermediate between broad-billed (*P. vittata*) and Antarctic (*P. desolata*) prions, evolved through homoploid hybrid speciation. MacGillivray’s prion (*P. macgillivrayi*), known from a single population on St Paul (Indian Ocean), has a bill width intermediate between *salvini* and *vittata* and could also be the product of interspecies introgression or hybrid speciation. Recently, another prion population phenotypically similar to *macgillivrayi* was discovered on Gough (Atlantic Ocean), where it breeds 3 months later than *vittata*. The similarity in bill width between the medium-billed birds on Gough and *macgillivrayi* suggest that they could be closely related. In this study, we used genetic and morphological data to infer the phylogenetic position and evolutionary history of *P. macgillivrayi* and the Gough medium-billed prion relative other *Pachyptila* taxa, to determine whether species with medium bill widths evolved through common ancestry or convergence. We found that Gough medium-billed prions belong to the same evolutionary lineage as *macgillivrayi*, representing a new population of MacGillivray’s prion that originated through a colonisation event from St Paul. We show that *macgillivrayi*’s medium bill width evolved through divergence (genetic drift) and independently from that of *salvini*, which evolved through hybridisation (gene flow). This represents the independent convergence towards a similarly medium-billed phenotype. The newly discovered MacGillivray’s prion population on Gough is of utmost conservation relevance, as the relict *macgillivrayi* population in the Indian Ocean is very small.

**Supplementary Information:**

The online version contains supplementary material available at 10.1007/s00438-021-01845-3.

## Introduction

Mayr (1963) affirmed that ‘‘Without speciation there would be no diversification of the organic world, no adaptive radiation, and very little evolutionary progress’’. Thus, investigating how species evolve is crucial to understand the evolutionary processes on Earth and the influence of speciation on both species’ persistence and patterns of species diversity (Seehausen et al. 2014). Given current Anthropocene biodiversity crisis, this could help develop effective ways to protect existing diversity and the diversification process itself (Rosenzweig 2001). A defining feature of species is that their populations are connected and integrity is maintained by gene flow (Rieseberg et al. 2004; Petit and Excoffier 2009), whereas reproductive barriers between species are important as they preserve adaptations (Mayr 1942). However, reproductive barriers are likely to be semipermeable to gene flow in recently diverged species or taxa in which hybrid incompatibilities evolve slowly, and thus speciation can occur in the presence interspecific gene flow (introgression) (Rieseberg et al. 2004; Seehausen et al. 2014). In line with this, Mayr’s (1963) notion that gene flow counteracts the process of speciation in animals is being eroded by examples of interspecific introgression, which can sometimes be adaptive (e.g. *Heliconius* butterflies, Nadeau et al. 2012), or occasional cases of homoploid hybrid speciation, where a fully reproductively isolated species can evolve if hybrid fitness is high (Nolte et al. 2006; Kunte et al. 2011; Lamichhaney et al. 2018). In most instances, interspecific introgression occurs between species that have diverged recently within the same adaptive radiation, because although such species have evolved distinct phenotypic traits, they are still sufficiently closely related to produce viable offspring (Seehausen et al. 2014; Masello et al. 2019).

One such adaptive radiation is that of the prions, *Pachyptila*, a genus of small petrels (Procellariidae) that differ primarily in bill size and structure (Warham 1990). Despite large oceanic distances separating breeding colonies, the prions are characterised by high levels of gene flow and incomplete reproductive isolation (Masello et al. 2019), which has blurred species limits resulting in the recognition of anything from three to nine *Pachyptila* species (Mathews 1934; Murphy 1936; Falla 1940; Fleming 1941; Fullagar 1972; Cox 1980; Harper 1980; Bretagnolle et al. 1990; Penhallurick and Wink 2004; Rheindt and Austin 2005; Howell and Zufelt 2019). Traditionally, most confusion has surrounded the larger-billed taxa (broad-billed prion *P. vittata*, average bill width, 21.4 mm, Salvin’s prion *P. salvini*, 17.1 mm, and Antarctic prion *P. desolata*, 14.3 mm), which have palatal lamellae on their upper mandibles adapted to filter zooplankton (Masello et al. 2019). The number of palatal lamellae is correlated with bill width, and these two variables determine the range of species that can be preyed upon (Masello et al. 2019). Palatal lamellae are best developed in *P. vittata*, allowing it to feed almost exclusively on copepods, whereas *P. desolata*, which feeds primarily on hyperiid amphipods, has less well-developed lamellae (Imber 1981; Bretagnolle et al. 1990; Klages and Cooper 1992; Cherel et al. 2002). The narrowest billed species (thin-billed prion *P. belcheri*, average bill width, 11 mm, fairy prion *P. turtur*, 11 mm; Masello et al. 2019) have only vestigial palatal lamellae and do not filter feed (Murphy 1936; Morgan and Ritz 1982; Bretagnolle et al. 1990; Klages and Cooper 1992). Since the larger-billed prions do not form a monophyletic clade, and all species except *P. belcheri* and *P. turtur* possess functional palatal lamellae, the most parsimonious explanation would be that both broad bills and lamellae represent the ancestral state in prions, with lamellae becoming vestigial once bill width reduced to below 12 mm (Masello et al. 2019). Murphy (1936) suggested that the thin bill of *P. belcheri* was structurally ancestral to that of *P. desolata*; however, the ancestral state of bill width in prions remains to be investigated. Genetically, *Pachyptila* comprises two distinct evolutionary clades, the first comprising the widest-billed species, *P. vittata,* and the other containing all other species (*P. turtur*, *P. belcheri*, *P. desolata*, and *P. salvini*) (Masello et al. 2019).

*Pachyptila salvini*, whose bill width is on average intermediate (17.1 mm) between *P. vittata* (21.4 mm) and *P. desolata* (14.3 mm), has the ability to feed on both copepods and hyperiid amphipods (Gartshore and Steele 1988; Ridoux 1994; Masello et al. 2019). Furthermore, although mitochondrial DNA (mtDNA) places *P. salvini* within the narrower-billed clade, coalescent simulations of microsatellite DNA showed that *P. salvini* evolved in a rare case of homoploid hybrid speciation between *P. vittata* and *P. desolata* (Masello et al. 2019). *Pachyptila salvini*’s intermediate bill width, with respect to *P. vittata* and *P. desolata*, allows it to feed on more prey species, giving it a potential feeding advantage over either of its parental species (Bretagnolle et al. 1990; Masello et al. 2019). Remarkably, *P. salvini*’s mid-summer breeding time is also intermediate between that of *P. vittata* (early summer) and *P. desolata* (late summer), thus isolating it reproductively, which might otherwise have led to the disappearance of its hybrid phenotype.

**Fig. 1:**
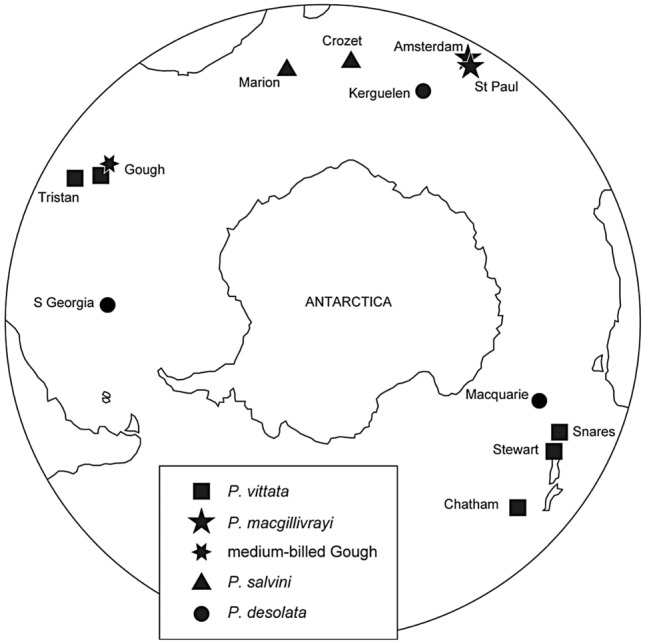
Locations of prions *Pachyptila* populations investigated around the Southern Ocean

MacGillivray’s prion (*P. macgillivrayi*) is another taxon with an average bill width (17.3 mm) intermediate between *P. salvini* and *P. vittata* (Roux et al. 1986). Although several authors recognise *P. macgillivrayi* as a species in its own right (Bretagnolle et al. 1990; Worthy and Jouventin 1999; Shirihai 2007), others have regarded it as a subspecies of either *P. salvini* (Roux et al. 1986) or *P. vittata* (Harper 1980). This little-known taxon comprises a single relict population confined to St Paul Island (38.7° S) in the southern Indian Ocean (Tollu 1984; Micol and Jouventin 2002; Jiguet et al. 2007), but it was formerly abundant on nearby Amsterdam Island (37.8° S, Worthy and Jouventin 1999; Fig. 1). *Pachyptila macgillivrayi* samples have been unavailable until recently, so the species was not included in the evolutionary genetic analysis of Masello et al. (2019). Its phylogenetic position with respect to narrower- and broader-billed clades (sensu Masello et al. 2019) and its microsatellite DNA allelic distribution are therefore unknown. Could the intermediate bill width of *P. macgillivrayi* also be a product of interspecies introgression, or even hybrid speciation?

Intriguingly, another prion population which is phenotypically similar to *P. macgillivrayi* was recently discovered on Gough Island in the South Atlantic Ocean, at similar latitude (40.3° S) to St Paul and Amsterdam islands in the Indian Ocean (Ryan et al. 2014). Gough Island is also home to the largest colony of *P. vittata*, which has disjunct South Atlantic (Tristan da Cunha and Gough) and southwest Pacific Ocean (New Zealand) populations (Fig. 1). On Gough Island, the recently discovered birds (hereafter Gough medium-billed) partially overlap in breeding distribution with *P. vittata*, but show a marked difference in bill width, with an average (18.5 mm; Ryan et al. 2014) that is also intermediate between *P. desolata* (14.3 mm) and *P. vittata* (21.4 mm; Masello et al. 2019). Given its similarity in bill width with *P. salvini* (17.1 mm) and *P. macgillivrayi* (17.3 mm; Roux et al. 1986; Masello et al. 2019), the Gough medium-billed population could be related to either species or represent a novel lineage. The Gough medium-billed prions breed roughly 3 months later than *P. vittata*, suggesting that they are a separate species (Ryan et al. 2014). Jones et al. (2020) showed that tracked *P. vittata* and Gough medium-billed individuals foraged and moulted in different areas of the South Atlantic Ocean, with the latter generally occurring farther south than *P. vittata*. Both the temporal and the latitudinal segregation have also been found in other prion species breeding in sympatry (Bretagnolle et al. 1990; Quillfeldt et al. 2015, 2020).

In this study, we report a morpho-genetic survey of *P. macgillivrayi* and both *Pachyptila* populations on Gough Island, and combine this with existing comparative data for other prion species to infer the phylogenetic position and evolutionary history of the Gough medium-billed prion, *P. macgillivrayi* and other *Pachyptila* taxa, given the poorly developed reproductive isolation in this genus, and to determine the ancestral state of bill width in prions.

## Materials and methods

### Samples and morphometric data

**Fig. 2:**
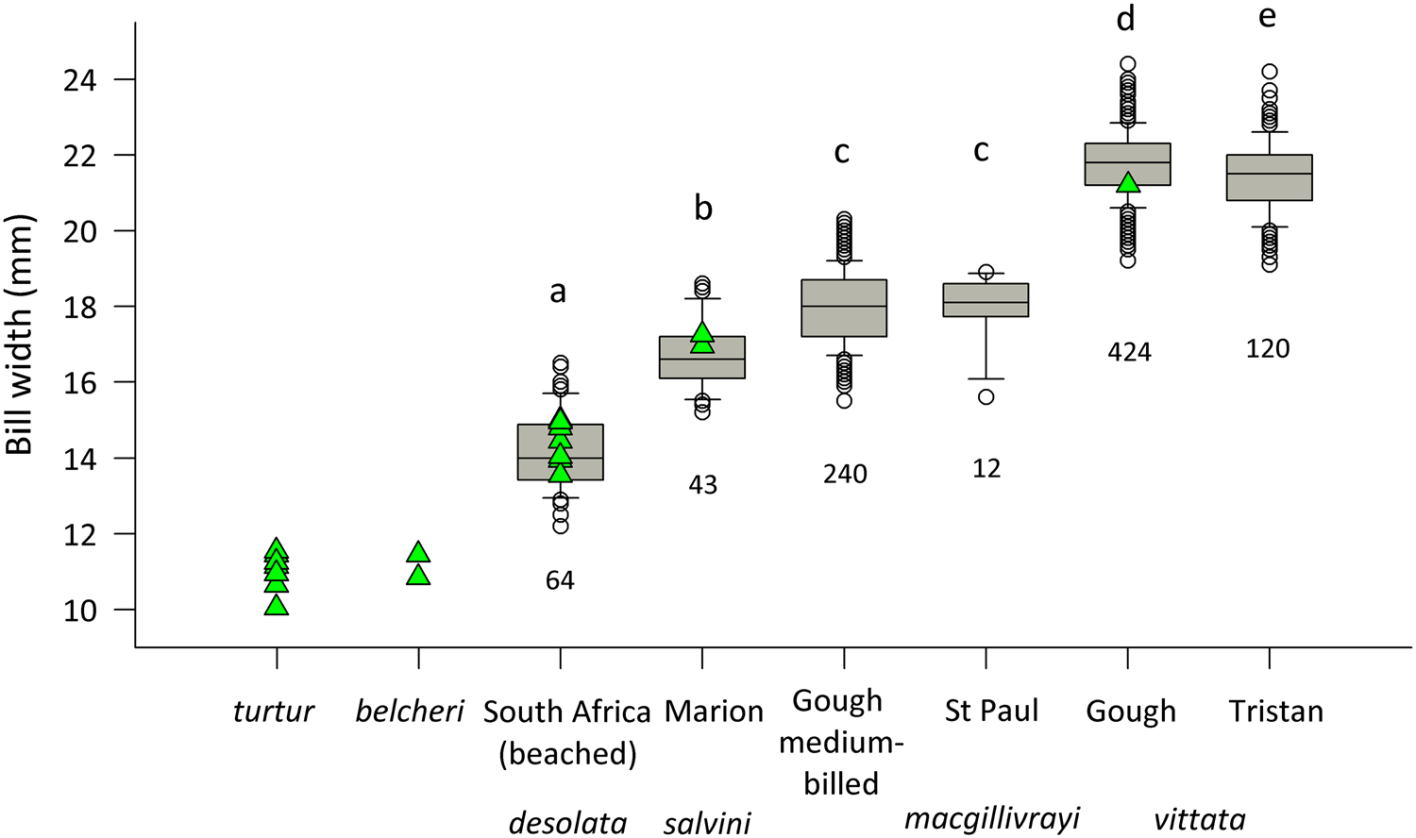
Differences in bill widths of adult larger-billed prions (*Pachyptila*). Box plot, Dunn’s homogenous subgroups (a–e), and sample sizes of the populations measured in this study. Box plots include medians, whiskers indicate variability outside the upper and lower quartiles (grey shaded block), and circles are outliers. Data from Masello et al. (2019) and references therein are shown here in green for comparison purposes; green triangles represent means for different colonies (*turtur*: 7; *belcheri*: 3; *desolata*: 7; *salvini*: 2; *vittata*: 1)

We first performed an analysis of average bill widths across populations of the larger-billed *Pachyptila* species, including Gough medium-billed (Ryan et al. 2014). We measured maximum bill widths of live or freshly dead adult prions to the nearest 0.1 mm on islands in the Tristan da Cunha archipelago (*P. vittata*), Gough Island (*P. vittata* and Gough medium-billed), St Paul Island (*P. macgillivrayi*), Marion Island (*P. salvini*), and beached specimens from South Africa (*P. desolata*; Fig. 1, for sample sizes see Fig. 2). We investigated differences in bill width using the Kruskal–Wallis test and Dunn's homogenous subgroups implemented in R (R Development Core Team 2019), as normality and equality of variance were not satisfied (*p* < 0.05).

For DNA analysis we collected muscle samples of *P. macgillivrayi* on St Paul Island (Fig. 1, Table 1). In addition, we sampled subfossil bones of *P. macgillivrayi* from Amsterdam Island (Table 1; Museum of New Zealand Te Papa Tongarewa registration numbers and GenBank accession numbers in Supplemental Material, Table S1). We also sampled both Gough medium-billed as well as *P. vittata* on Gough Island (Fig. 1, Table 1). To ensure we sequenced individuals from the correct populations, samples from Gough were taken from birds found outside the range of morphological overlap, which is roughly a bill width between 19.5 and 20.5 mm (Ryan et al. 2014). Therefore, all *P. vittata* sampled had bills > 21 mm wide and were collected from the area around the meteorological station where *P. vittata* predominates, and all Gough medium-billed birds had bills < 19 mm and were collected from Gonydale, a highland valley where medium-billed birds occur. To place our newly sampled populations in broader context, we compared their DNA data to previously sequenced or genotyped samples (Masello et al. 2019), and to new samples from other taxa. These additional samples were collected from birds at different breeding localities (Fig. 1) between 1999 and 2014, including *P. vittata* from Tristan da Cunha (Nightingale Island), islets off Stewart Island, Snares Island and the Chatham Islands (Rangatira Island) off New Zealand; *P. salvini* from the Prince Edward Islands (Marion Island) and the Crozet Islands; *P. desolata* from Kerguelen, South Georgia and Macquarie Island; *P. turtur* from Mana Island (New Zealand), and the closely related blue petrel (bird wrecked at New Zealand) to be used as outgroup (Table 1). Most DNA samples were extracted from fragments of muscle or skin tissue collected from fresh prion corpses, but some feathers and blood samples were taken from live birds (Table 1). Tissue samples were stored in ethanol, dry as feather quills, blood in Queens’s lysis buffer (Kerguelen) and blood on FTA classic cards (Whatman International Ltd, Maidstone, UK; Chathams, South Georgia, Macquarie). Some carcasses were stored frozen prior to sampling. Samples sizes are provided in Table 1.

**Table 1:** Prion *Pachyptila* sequences and genotypes analysed

Taxa	Location	Sample type	*n* Sequenced for mtDNA	GenBank accession numbers		*n* Genotyped, 18 microsatellite loci
				Cytochrome *b*	COI	
*vittata*	Gough Island	Muscle	5	KX139098-102^a^	KX092029, 30, 33, 35, MT881654^b^	52^a^
	Tristan (Nightingale Island)	Muscle	5	KX139103, 107–08, 111–12^a^	KX092024-28^b^	36^a^
	Chatham (Rangatira)	Blood	3	MF159025-7^b^	KX092015-7^b^	30^a^
	Stewart Island	Muscle	6	MF159020-21, 23, MF167647-49^b^	KX092018-23^b^	
	Snares Island	Muscle	2	MF159028-29^b^	KX092036-7^b^	
*salvini*	Marion Island	Feather quill	3	KX139067-69^a^	KX092041-43^b^	18^a^
	Crozet Islands	Muscle	1	KX139077^a^	KX092044^b^	
*macgillivrayi*	St Paul Island	Muscle	10	KX139078-83, 85–88^b^	KX092045, 47–48, 50, 52–57^b^	12^b^
	Amsterdam Island	Subfossil bone	1	MF159022^b^	KX092069^b^	
Medium-billed	Gough Island	Muscle	6	KX139089, 91–95^a^	KX092060-65^b^	10^b^
*desolata*	Kerguelen	Blood	1	KX139130^a^	KX092013^b^	38^a^
	South Georgia	Blood				35^a^
	Macquarie Island	Blood				7^a^
*belcheri*	New Zealand (beached)	Muscle	1	MF159024^b^	KX092014^b^	
*turtur*	New Zealand	Blood	2	MZ054169, MZ268100^b^	MZ268116^b^, MK262522.1^c^	
*Halobaena caerulea*	New Zealand	Muscle	1	MZ054168^b^	MK261916.1^c^	

^a^Previously sequenced or genotyped for Masello et al. (2019)

^b^Sequenced for this study

^c^Obtained from GenBank

### Molecular methods

For the subfossil bones a ~ 5 mm fragment of bone was removed from the broken ends of *humeri* using a Dremel grinder with a new Dremel wheel used for each bone. Bone samples were powdered by grinding in sterilised mortars and pestles. Bone powder was then decalcified and a phenol–chloroform extraction performed (Shepherd and Lambert 2008) to isolate the DNA.

DNA was extracted from modern samples using a Qiagen DNeasy^®^ Blood and Tissue kit (Qiagen, Germany), following the manufacturer’s instructions. The final elution volume was 100 µl for blood and modern tissue extractions and 45 µl for feather samples. Extractions and PCR setups from subfossil bones were performed in a dedicated ancient DNA (aDNA) laboratory located in a different building from where modern DNA and PCR products were handled. Potential contamination was monitored by the use of negative extraction and PCR controls.

The mitochondrial cytochrome *b* (cyt *b*) gene has previously provided a reasonable estimate of maternal evolutionary relationships among a range of *Pachyptila* species, which were largely congruent with data from nuclear microsatellites (Moodley et al. 2015) and nuclear introns (Masello et al. 2019). We therefore used this marker, as well as sequence data from the cytochrome *c* oxidase subunit 1 (COI), to reconstruct maternal relationships among these taxa using a subset of 44 samples (Table 1). An 811-base pair (bp) fragment of cyt *b* was PCR amplified using specific primers (CytB_Pri_F: 5′-CTAGCTATACACTACACCGC-3′ and CytB_Pri_R: 5′-CTAGTTGGCCGATGATGATG-3′) (Moodley et al. 2015). Primer3 (Untergasser et al. 2012) was used to design novel internal primers for amplifying cyt *b* in ancient DNA samples. These primers amplified five overlapping fragments which ranged in size from 209 to 269 bp (CytB_Pri_F with Pricytbint1R: 5′-AGGATGACTCCTGTGTTTCATGT-3′; Pricytbint2F: 5′-CCACATTGGACGAGGACTTT-3′ with Pricytbint2R: 5′-GGCAAAGAATCGGGTTAGTG-3′; Pricytbint3F: 5′-CCCTCGTAGAATGAGCCTGA-3′ with Pricytbint3R: 5′- GGGGGAGAATAGGGCTAAAG-3′; Pricytbint4F: 5′- CGGCATCGTATCAAACTGTG-3′ with Pricytbint4R: 5′- TTGAGCGTAGGATGGCATAA-3′ and Pricytbint5F: 5′-YCCTCCCCATATTAAACCAGA-3′ with CytB_Pri_R). A 648 bp fragment of cytochrome *c* oxidase subunit I (COI) was amplified from the modern samples using the AWCF1 and AWCR6 primers of Patel et al. (2010). Ancient DNA samples were amplified and sequenced from short overlapping fragments of COI using internal primers from Patel et al. (2010).

PCR amplifications of cyt *b* were performed in 20 μl reaction volumes containing 100 ng DNA template, 10 mM of each primer, 10 mM dNTPs (Roth, Karlsruhe), 2 mM MgCl and 5 U Taq DNA polymerase (BioLabs Taq DNA polymerase) in a 1 × PCR reaction buffer. Thermocycling conditions involved an initial denaturation at 94 °C for 2 min, 30 cycles of denaturation at 94 °C for 30 s, annealing at 60 °C for 45 s and extension at 72 °C for 1 min, followed by a final extension step of 5 min at 72 °C. PCRs for COI were conducted in 10 μl volumes containing 1 × PCR buffer, 200 μM of dNTP, 0.5 U of Taq DNA polymerase (Roche), 0.3 M of BSA and 0.5 µM of primer. Thermocycling involved initial denaturation of 2 min at 94 °C, followed by 35 cycles of 94 °C for 30 s, 50 °C for 40 s and 72 °C for 1 min, followed by a final extension of 10 min at 72 °C.

PCR products were purified by digestion with exonuclease-shrimp alkaline phosphatase (from USB Corp, Cleveland for COI and Fermentas Life Sciences for cyt *b*), following the manufacturer’s specifications. PCR products were then sequenced in both directions using Big Dye chemistry (Applied Biosystems) and run on an AB 3130xl genetic analyser (for cyt *b*) or ABI3730 (for COI). Cyt *b* sequences were assembled and aligned in CLC Main Workbench 6.9.2 (CLC bio, Aarhus, Denmark). COI sequences were edited in Sequencer 5.2.3 (Gene Codes Corporation), and were aligned manually as they contained no indels.

Twenty-five previously isolated prion microsatellite loci (Moodley et al. 2015) were also amplified from genomic DNA of the St Paul *P. macgillivrayi* and Gough medium-billed samples. These were run together with samples of known genotype, so that these data may be calibrated against those of other prion microsatellite studies. Microsatellite profiles were checked for null alleles using MICROCHECKER (van Oosterhout et al. 2004) and for deviation from genotypic equilibrium (Hardy Weinberg equilibrium, HWE) using FSTAT (Goudet 1995). Multiple tests were corrected for using a Bonferroni correction. For context, these newly generated microsatellite data were analysed together with 216 samples of *P. vittata* from Gough, Tristan and Chathams, *P. salvini* from Marion, and *P. desolata* from Kerguelen, South Georgia and Macquarie (Table 1), published in previous studies (Moodley et al. 2015; Quillfeldt et al. 2017; Masello et al. 2019).

### Genetic diversity and structure

Genetic diversity parameters were estimated for cyt *b* and COI for each population with at least five samples using DnaSP v5 (Librado and Rozas 2009) (Table 2). We estimated also two demographic indicators, Tajima’s *D* (1989) and Fu’s Fs (1997) (Table 2), for each gene and each species/population using DnaSP v5. For microsatellites, the allelic richness (*AR*, mean number of alleles per locus) was estimated using GENETIX 4.05 (Belkhir et al. 2004) and rarefied for differences in sample size using ADZE 1.0 (Szpiech et al. 2008). Unbiased expected heterozygosity (*H*_E_) and observed heterozygosity (*H*_O_) were also estimated in GENETIX.

**Table 2:** Genetic diversity and population demography for cytochrome *b* (cyt *b*) and cytochrome c oxidase subunit I (COI) sequences from prion *Pachyptila* taxa (only populations with at least 5 samples are presented)

	Diversity					Demography	
	*n*	*P*	*K*	HD	*π*	*D*	*F*_S_
Cyt *b* (812 bp)							
*macgillivrayi*, St Paul	10	5	1.000	0.533	0.001	− 1.741*	− 0.876^ns^
Gough medium-billed	6	3	1.000	0.800	0.001	− 1.233^ns^	− 1.813^ns^
*vittata*, Gough	5	2	0.800	0.700	0.001	− 0.973^ns^	− 0.829^ns^
*vittata*, Tristan	5	0	0	0	0	–	–
*vittata*, Stewart	6	0	0	0	0	–	–
COI (774 bp)							
*macgillivrayi*, St Paul	10	3	0.600	0.378	0.001	− 1.562^ns^	− 0.459^ns^
Gough medium-billed	6	5	2.067	0.733	0.003	− 0.315^ns^	1.081^ns^
*vittata*, Gough	5	4	1.600	0.700	0.002	− 1.094^ns^	0.276^ns^
*vittata*, Tristan	5	5	2.200	0.700	0.003	− 0.562^ns^	0.804^ns^
*vittata*, Stewart	6	5	2.467	0.933	0.003	− 0.351^ns^	− 1.672^ns^

	*n*	AR	PR	*H*_E_	*H*_O_
Microsatellites					
*macgillivrayi*, St Paul	12	5.7	0.562	0.694	0.684
Gough medium-billed	10	6.4	1.002	0.737	0.707
*vittata*, Gough	52	8.3	0.412	0.726	0.701
*vittata*, Tristan	36	6.9	0.423	0.689	0.614
*vittata*, Chatham	30	6.5	0.323	0.662	0.614

See also Table 1

*n* number of individual samples, *P* number of polymorphic (segregating) sites, *K* average number of pairwise differences, *HD* haplotype diversity, *π* the nucleotide diversity, *AR* allelic richness, *PR* private allelic richness, *D* Tajima’s statistic, *F*_*S*_ Fu’s statistic

Statistical significance: *P < 0.05; ^ns^not significant

Maternal genetic structure among prion species was determined using two methods. First, we concatenated both mtDNA genes and generated a median-joining network (Bandelt et al. 1999) of the composite haplotypes in POPART (Leigh and Bryant 2015). Then, we used the same DNA sequence data to reconstruct phylogenetic relationships using a Bayesian multispecies coalescent framework in BEAST 2 (Bouckaert et al. 2014). The best nucleotide substitution model was determined for each of the two alignments using JMODELTEST (Posada 2008). Site models were thus unlinked for each partition. Each gene tree was unlinked, and a species tree using a birth–death model prior was used to account for gene tree incongruence. The population prior allowed for changes in effective population size, but assumed a constant ancestral population size. All clock models were unlinked, but to determine the correct clock prior, alternative models assuming lognormal and exponential priors were tested against a strict molecular clock for each partition. A soft-bounded lognormal prior (*α* = 1.2, *β* = 1.0) of no later than 5 million years for the emergence of the genus was based on the earliest fossil evidence (Olson 1983, 1985a, b). After a single run of 100,000,000 MCMC iterations, sampling every 100,000 steps and discarding 20% as burn-in, the standard deviations of the posterior marginal distributions of both these parameters included zero in all cases, making them no more likely than a null strict clock for all gene partitions. All subsequent analyses were therefore carried out assuming a strict clock. Since we were unsure of how *P. macgillivrayi* and Gough medium-billed population were related to other prions, we also included two samples of taxa with vestigial lamellae (*P. belcheri* and *P. turtur*), as well as *H. caerulea* as outgroup (Table 1), to help identify the narrower-billed clade in both network and phylogenetic analyses.

Nuclear genetic structure was assessed using the Bayesian clustering algorithm implemented in STRUCTURE 2.3.4 (Pritchard et al. 2000). We assumed an admixture model because our previous molecular analysis of prions (Masello et al. 2019) showed consistent evidence of incomplete reproductive isolation and secondary contact. Moreover, inter-island movements have been recorded among well-studied procellariiforms (e.g. wandering albatross *Diomedea exulans*; Inchausti and Weimerskirch 2002), as well as a lack of genetic structure in many oceanic pelagic species in the Southern Ocean (e.g. Burg and Croxall 2001), including some Atlantic and Indian Ocean *Pachyptila* populations (Quillfeldt et al. 2017). The analysis was run ten times for K1–10 with each run randomly started, consisting of 500,000 Markov Chain Monte Carlo (MCMC) iterations, assuming correlated allele frequencies and removing the first 100,000 runs as burn-in. The mean likelihood values across multiple values of K were determined using STRUCTURE HARVESTER (Earl and vonHoldt 2012) in accordance with Evanno’s method (Δ*K,* i.e. the rate of change in the log probability of data between successive *K* values) (Evanno et al. 2005). Assignment plots were constructed for all values of *K* that were biologically interpretable. We used CLUMPAK (Kopelman et al. 2015) for the creation of genetic ancestry figures. We also used ARLEQUIN 3.5 (Excoffier and Lischer 2010) to implement analyses of molecular variance (AMOVAs) on the microsatellite data. We tested several a priori groupings of populations, with the expectation that the variance component distributed between populations (*F*_ST_) and among defined groups (*F*_ct_) would be highest for the evolutionarily correct grouping. The significance of F statistics and variance components were tested with 99,999 permutations. We also calculated pairwise *F*_ST_ (after Weir and Cockerham 1984) between all populations in ARLEQUIN 3.5 (Excoffier and Lischer 2010), with significance again determined with 99,999 permutations.

Given previously observed levels of introgression in prion species (Masello et al. 2019), we determined the proportion of genetic variation within *P. macgillivrayi* and Gough medium-billed prions that was derived through introgression using Bayesian inference. Since we were only interested in gene flow into and out of the two newly sampled medium-billed populations, we tested for the presence of bi-directional migration between *P. macgillivrayi* and Gough medium-bills, as well as between both these populations and Gough’s sympatric *P. vittata* population. We inferred gene flow using BAYESASS 3.0 (Wilson and Rannala 2003), which estimates the posterior probability of an individual’s history and allows an estimation of the rate and direction of recent dispersal (Genovart et al. 2013). The acceptance rates for the main parameters (i.e. ‘migration’ rate, inbreeding coefficient and allele frequencies) were adjusted during preliminary runs. Convergence was evaluated by inspection of the trace files in TRACER 1.5 (Rambaut et al. 2018). Final parameter estimates were attained after performing three independent runs by means of different starting random seed numbers. The MCMC was run for 50,000,000 iterations with a burn-in period of 10,000,000 and a sampling frequency of 5000 iterations.

### Reconstruction of ancestral bill state

To understand the evolution of bill width among *Pachyptila* species, we reconstructed the ancestral state of bill width using BAYESTRAITS v3.0.2 (Pagel et al. 2004). We used bill width data collected in the present study for larger-billed species, together with mean bill width of *P. turtur* and *P. belcheri* from Masello et al. (2019), and the phylogenetic tree (topology and branch lengths) reconstructed above. We also measured bill width of blue petrels *Halobaena caerulea* from South Georgia and Kerguelen (Supplemental Material, Table S2). Since this trait varied continuously among taxa, we selected the “Continuous: Random Walk” option (Pagel 1999, Model A), which uses a generalised least squares model to reconstruct the posterior distribution of bill width at the ancestral node of the *Pachyptila* tree. We accounted for phylogenetic uncertainty by using the entire post-burn-in posterior sample of trees generated by BEAST 2. We then inferred posterior distribution of ancestral bill widths using MCMC iterations, with a chain length of 1,001,000 iterations and a burn-in of 200,200 iterations. We performed this analysis with and without the outgroup *H. caerulea* to examine whether the ancestral state of bill width changed since the genera *Pachyptila* and *Halobaena* shared a common ancestor.

### Additional comparisons

We also report variation in bill colour among the prions at Tristan and Gough, to investigate its usefulness for distinguishing among *Pachyptila* taxa, given the assertion that *P. vittata* differs from all other prions in having a blackish bill (e.g. Marchant and Higgins 1990; Shirihai 2007).

## Results

Bill width varied significantly among the larger-billed prion taxa measured in this study (Kruskal–Wallis, *χ*^2^ = 679.5, *df* = 5, *P* ≤ 0.001). An average bill width of 14.2 mm was found in *P. desolata*, 16.7 mm in *P. salvini*, 18.0 mm for both Gough medium-billed and *P. macgillivrayi*, and 21.8 and 21.4 mm for *P. vittata* from Gough and Tristan, respectively (Fig. 2, and Supplemental Material, Table S2). Dunn’s homogenous subgroups revealed that the Gough medium-billed prions were similar to *P. macgillivrayi* (homogeneous subgroup ‘c’), and that both taxa were significantly different from all other prion taxa (Fig. 2, and Supplemental Material, Table S3).

The final mtDNA alignment, including outgroup sequences, comprised 811 bp of cyt *b* sequence and 648 bp of COI sequence from 47 individuals. This alignment also included a sequence from a subfossil *P. macgillivrayi* bone from the extinct Amsterdam Island population (S.34710.1) that produced full-length COI and cyt *b* sequences (Table 1, and Supplemental Material, Table S1). The COI sequence obtained for this sample was identical to the most common haplotype detected from modern *P. macgillivrayi* sampled on St Paul Island and the cyt *b* sequence differed from the most-closely related sequence by two substitutions. Only a 231 bp COI sequence could be obtained from the Amsterdam Island *P. macgillivrayi* bone specimen S.35077.1 and this was identical to the sequence from S.34710.1 and was not used in further analyses (Supplemental Material, Table S1).

Genetic diversity was higher for COI than for cyt *b* (Table 2), with *P. macgillivrayi* also featuring a higher relative diversity at the former locus and a lower diversity at the latter. Both loci showed negative values for Tajima’s *D* and Fu’s Fs which, assuming mitochondrial neutrality, may indicate signatures of recent population expansions, although only the *P. macgillivrayi* population from St Paul Island showed significantly negative Tajima’s *D* for COI (Table 2). Average microsatellite diversity indices showed equally high values for the investigated populations, with relatively higher values for *P. vittata* from Gough Island (Table 2). Private allelic richness was highest among the Gough medium-billed prions but, in general, was relatively low (Table 2).

**Fig. 3:**
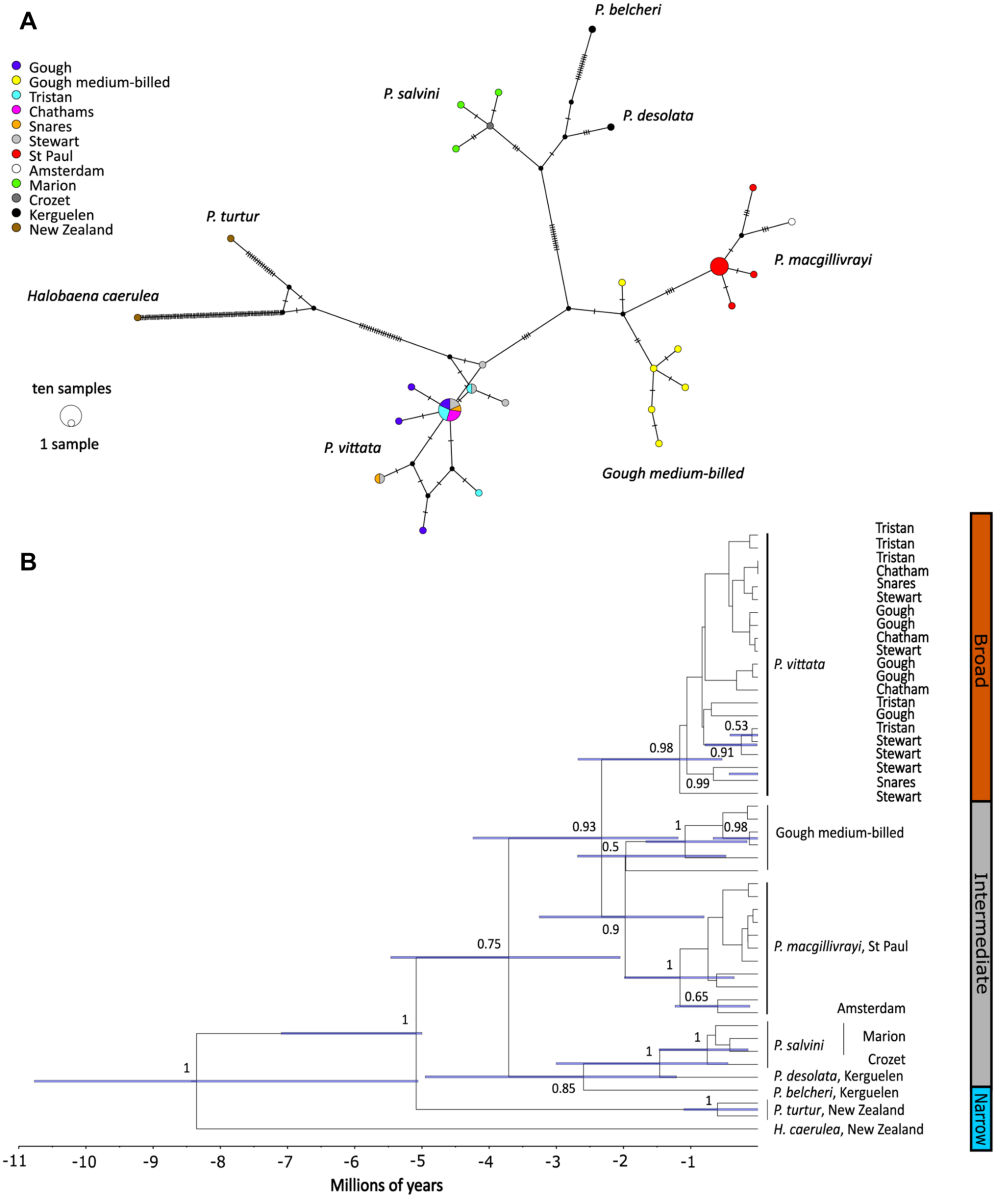
Medium-joining haplotype network (**A**) and inferred Bayesian phylogeny (**B**) based on the combined sequence data for cyt *b* and COI for the prion taxa (*Pachyptila*) in this study. In the network, the circle size is proportional to haplotype frequency, hash marks crossing line connections represent mutational steps, and nodes without circles correspond to hypothetical haplotypes not sampled. In the inferred phylogeny, for clarity reasons, 95% HPD bars and probabilities are only shown for nodes with posterior probabilities > 0.5

The maternal phylogenetic relationships among prion taxa inferred from the median-joining haplotype networks and the Bayesian species tree (Fig. 3) identified the two previously detected broader- and narrower-billed clades (Masello et al. 2019), with the narrow-billed *P. turtur* diverging basally some 5–7.1 Mya (95% highest posterior density, HPD). *Pachyptila macgillivrayi* samples from St Paul and Amsterdam Island formed a monophyletic clade. This clade was sister to another monophyletic clade made up exclusively of Gough medium-billed prions, which appears to have diverged from other *P. macgillivrayi* about 0.8–3.3 Mya (95% HPD, Fig. 3B). Interestingly, this monophyletic *P. macgillivrayi*-Gough medium-billed clade was not closely related to *P. salvini*, as was expected from their similar intermediate bill widths (Fig. 2), instead clustering within the broad-billed evolutionary group, sister to *P. vittata* (Fig. 3B).

**Fig. 4:**
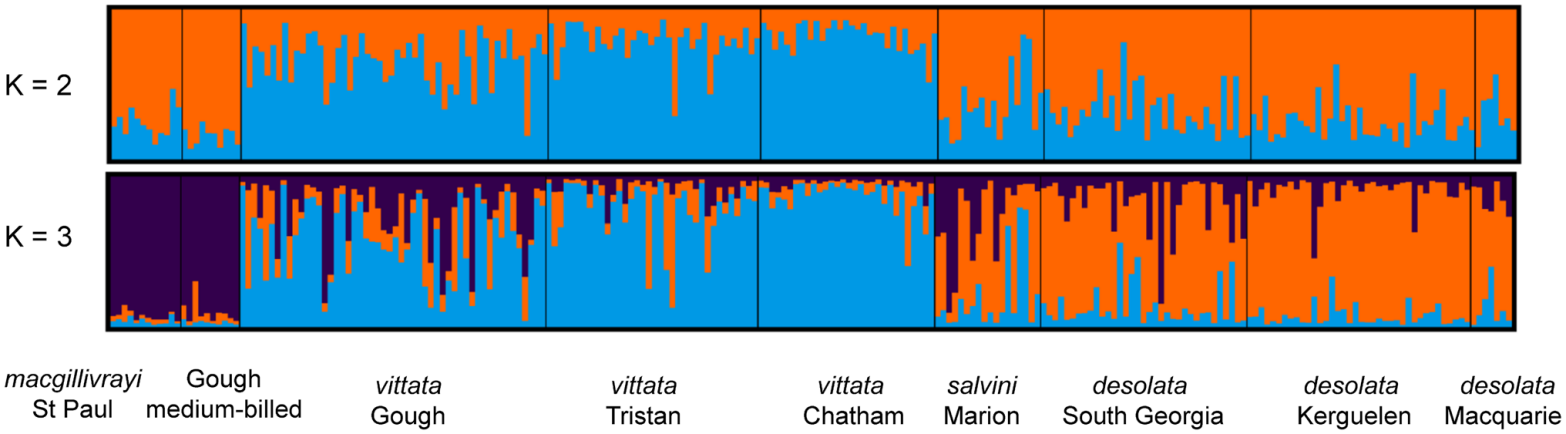
Genetic ancestry based on an 18 microsatellite loci data set for the prion taxa (*Pachyptila*) in this study. Genetic structure was modelled in STRUCTURE assuming admixture and the partitioning of genetic diversity into two (*K* = 2) and three (*K* = 3) populations (K4–10 shown in Supplemental Fig. S1). Each line on the plot represents an individual multilocus profile

This mtDNA structure was broadly reflected in the microsatellite data (Fig. 4 and Supplemental Material, Fig. S1 for K1–10). After STRUCTURE analysis, the Evanno method (Evanno et al. 2005) estimated the highest Δ*K* (101.5) for *K* = 2, in which narrower- and broader-billed groups were separated. In contrast to mtDNA, *P. macgillivrayi* and Gough medium-billed prions partitioned with narrower-billed species at *K* = 2, however, at *K* = 3, both formed their own cluster, distinct from both narrower- and broader-billed groups. Structure analyses also revealed that the multilocus allele profiles of *P. vittata* from Gough appear admixed with *P. macgillivrayi* alleles (Fig. 4).

We further tested the structure of populations by analysing the molecular variance in our microsatellite data set in an AMOVA framework. Among the various grouping scenarios tested, the structure separating *P. macgillivrayi* from St Paul Island from all other populations returned the highest among group variance (*F*_ct_). This value was only slightly lower when Gough medium-billed prions were included in the same group as *P. macgillivrayi* (Table 3). Including Gough *P. vittata* into this group returned a much lower *F*_ct_ value. These results are further supported by pairwise *F*_ST_ values among populations, which were lowest between *P. macgillivrayi* and Gough medium-billed prions, higher between *P. macgillivrayi* and Gough *P. vittata* and highest in comparisons to the other *P. vittata* populations (Table 4).

**Table 3:** Analysis of molecular variance (AMOVA) performed on 18 microsatellite loci from prion *Pachyptila* taxa

Genetic structure tested	Source of variation	Degrees of freedom	Sum of squares	Variance component	Percentage of variation	*F*_sc_	*F*_ct_
1 Group: 1 (no structure)	Among populations	4	43.974	0.147***	4.18	–	–
Group 1: *macgillivrayi* St Paul, Gough medium-billed, *vittata* Gough, *vittata* Tristan, *vittata* Chatham	Within population	275	929.833	3.381	95.82		
	Total	279	973.807	3.52			
2 Groups:	Among groups	1	15.075	0.198^ns^	5.36	0.030***	0.054^ns^
Group 1: *macgillivrayi* St Paul	Among populations within groups	3	28.898	0.105***	2.86		
Group 2: Gough medium-billed, *vittata* Gough, *vittata* Tristan, *vittata* Chatham	Within populations	275	929.833	3.381***	91.77		
	Total	279	973.807	3.684			
	Among groups	1	10.046	0.095^ns^	2.63	0.037***	0.026^ns^
Group 1: Gough medium-billed	Among populations within groups	3	33.928	0.130***	3.62		
Group 2: *macgillivrayi* St Paul, *vittata* Gough, *vittata* Tristan, *vittata* Chatham	Within populations	275	929.833	3.381***	93.76		
	Total	279	973.807	3.606			
	Among groups	1	19.718	0.185^ns^	5.09	0.023***	0.051^ns^
Group 1: *macgillivrayi* St Paul, Gough medium-billed	Among populations within groups	3	24.256	0.081***	2.21		
Group 2: *vittata* Gough, *vittata* Tristan, *vittata* Chatham	Within populations	275	929.833	3.381***	92.70		
	Total	279	973.807	3.648			
	Among groups	1	20.816	0.074^ns^	2.09	0.028***	0.021^ns^
Group 1: *macgillivrayi* St Paul, Gough medium-billed, *vittata* Gough	Among populations within groups	3	23.158	0.097***	2.74		
Group 2: *vittata* Tristan, *vittata* Chatham	Within populations	275	929.833	3.381***	95.17		
	Total	279	973.807	3.553			
3 Groups:	Among groups	2	34.688	0.130***	3.34	0.011**	0.033^ns^
Group 1: *macgillivrayi* St Paul, Gough medium-billed	Among populations within groups	2	11.013	0.041*	1.06		
Group 2: *vittata* Gough	Within populations	275	1021.781	3.716***	95.60		
Group 3: *vittata* Tristan, *vittata* Chatham	Total	279	1067.482	3.886			

*N* individuals in each population: *macgillivrayi* St Paul (12), Gough medium bill (10), *vittata* Gough (52), *vittata* Tristan (36), *vittata* Chatham (30)

*P*-values: ***P < 0.001, **P < 0.01, *P < 0.05, ^ns^not significant (*P* > 0.05)

**Table 4:** Estimates of *F*_ST_ from microsatellites pairwise values (below diagonal) and average number of uncorrected pairwise differences from prion *Pachyptila* taxa

	*macgillivrayi* Saint Paul	Gough medium-billed	*vittata* Gough	*vittata* Tristan	*vittata* Chatham
*macgillivrayi*, Saint Paul	–	7.590	7.540	7.126	7.682
Gough medium-billed	0.034**	–	7.775	7.271	7.780
*vittata*, Gough	0.052***	0.038***	–	6.722	7.145
*vittata*, Tristan	0.106***	0.078***	0.020***	–	6.229
*vittata*, Chatham	0.123***	0.092***	0.031***	0.006^ns^	–

*P* values: ***P < 0.001, **P < 0.01, *P < 0.05, ^ns^not significant (*P* > 0.05)

Using BAYESASS, we estimated bidirectional interspecific migration rates, which were generally low, except in the case of the Gough medium-billed prions, where up to 28% of that gene pool could have been derived through unidirectional gene flow from *P. macgillivrayi* from St Paul, and up to 8% derived from Gough *P. vittata* (Table 5; results for all studied populations are provided in the Supplemental Material, Table S4).

**Table 5:** Posterior mean migration rates and standard deviation of the marginal posterior distribution for each estimate. Mean migration rates (m) as a proportion from 0 to 1 and standard deviation (SD)

Pop. 1\Pop. 2	*macgillivrayi* St Paul	Gough medium-billed	*vittata* Gough
*macgillivrayi* St Paul	–	0.0270 (0.0250)	0.0312 (0.0285)
Gough medium-billed	0.2815 (0.0383)	–	0.0766 (0.0476)
*vittata* Gough	0.0070 (0.0068)	0.0064 (0.0063)	–

Values below the diagonal correspond to *m* [1][2] (± SD) which is the fraction of individuals in population 1 (column far left) that are migrants derived from population 2 (top line) per generation. Values above the diagonal correspond to *m* [2][1] (± SD)

**Fig. 5:**
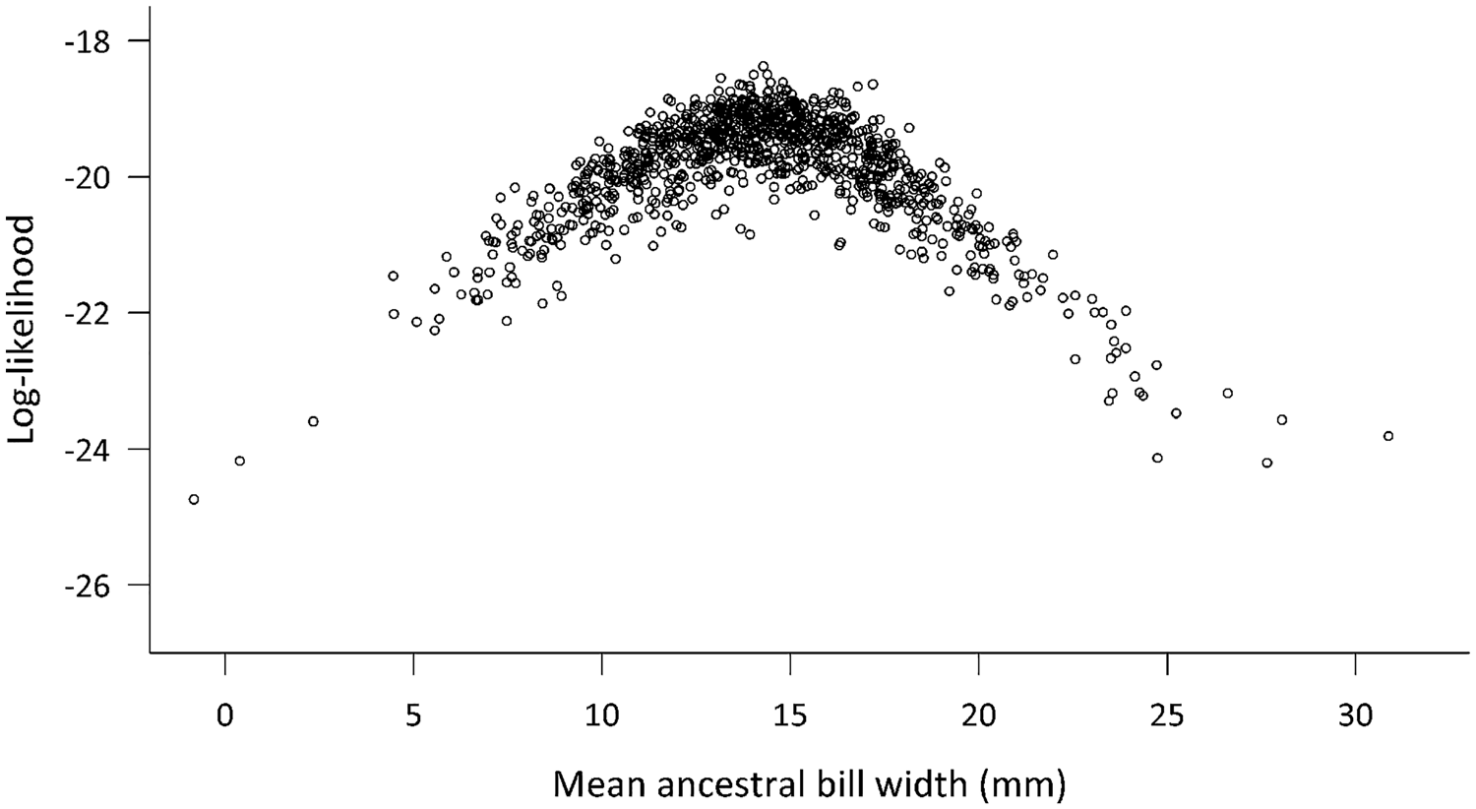
Ancestral state reconstruction of bill width at the root of the *Pachyptila* phylogeny. This posterior distribution of ancestral bill widths was inferred as a continuously varying trait using generalised least squares in BAYESTRAITS

The most likely bill width in the common ancestor to all extant *Pachyptila* species was 14.2 mm (median posterior ancestral bill width estimated by BAYESTRAITS; Q1 = 11.9, Q3 = 16.4; Fig. 5). Including *Halobaena caerulea*, an even narrower ancestral median bill width of 12.8 mm was inferred (Q1 = 8.7, Q3 = 17; Supplemental Material, Fig. S2).

Bill colour varies considerably among prions at Gough and Tristan (Supplemental Material, Fig. S3). Although many *P. vittata* have blackish bills (Fig. S3A, B) similar to this taxon in New Zealand (Marchant and Higgins 1990; Shirihai 2007, but see Supplemental Material, Fig. S4), some have bluish-grey sides to the bill, even among birds breeding in spring at Inaccessible Island, Tristan da Cunha (Fig. S3C), and one unusual bird has been photographed on Gough with bright blue sides to the bill (Fig. S3D). Gough medium-billed birds seemingly show less variation, with generally lead-grey bills with variably paler blue-grey bill sides and tips (Fig. S3E, F). However, blackish bills are also not confined to *P. vittata*, as some fairly small-billed birds (likely *P. desolata*) photographed at sea near the Crozets in December 2016 had blackish bills (Supplemental Material, Fig. S5A, B) compared to typical *P. desolata* (Fig. S5C, D). Consequently, bill colour was not a reliable trait for distinguishing these taxa.

## Discussion

### Are Gough Island medium-billed prions an undiscovered population of *P. macgillivrayi*?

The medium-billed prions from Gough Island clustered in a monophyletic clade, which was sister to *P. macgillivrayi* (Fig. 3), and microsatellite genotypes from 10 Gough medium-billed individuals consistently clustered them with *P. macgillivrayi* at *K* ≥ 3 (Fig. 4). These results suggest that the Gough medium-billed prions belong to the same evolutionary lineage as *P. macgillivrayi* with which it shared a common ancestor between 0.8 and 3.3 Mya. The use of only mtDNA and a single fossil calibration could potentially also have led to biases in divergence times, and although the relative branching patterns are independent of divergence time, we suggest the reader consider the full 95% HPD when interpreting the timing of evolutionary events. Despite this potentially old divergence, microsatellite variation among Gough medium-billed prions did not differ from that of *P. macgillivrayi.* This suggests that Gough medium-billed prions represent a hitherto undiscovered population of MacGillivray’s prion. These results also imply that the *P. macgillivrayi* medium-billed phenotype must have evolved prior to the Pleistocene divergence of the Gough medium-billed population.

### Colonisation of Gough Island by *P. macgillivrayi*

Our analysis of gene flow among *P. macgillivrayi*, Gough medium-billed prions and *P. vittata* confirmed admixture analyses in STRUCTURE that indicated low levels of gene flow into the *P. macgillivrayi* population on St Paul Island. However, the same analysis revealed that a significant proportion of the Gough Island medium-billed gene pool stemmed from St Paul *P. macgillivrayi*. This could represent ongoing gene flow between these two populations, or it could be the molecular signature of the original colonisation event that brought medium-billed prions to Gough Island. However, despite Gough medium-billed population being much larger (875,000 breeding pairs; Caravaggi et al. 2019) than the *P. macgillivrayi* population on St Paul (a few hundred birds; Tollu 1984; Shirihai 2007), the inferred migration into the Gough medium-billed population was unidirectional, which is more compatible with a colonisation event as ongoing gene flow is more likely to result in bi-directional gene flow. On the other hand, we also detected lower levels of unidirectional gene flow from Gough *P. vittata* into the Gough medium-billed population (Table 5), which is supported by their low pairwise *F*_ST_ value of 0.038 (Table 4). This incoming gene flow from *P. vittata* may also explain the overlap in bill widths among the smallest *P. vittata* and the largest Gough medium-billed prions (Fig. 2). Because these populations are sympatric, breeding on the same island (Ryan et al. 2014; Jones et al. 2020), this result is likely to represent ongoing gene flow, with directionality determined by assortative mating of medium-billed females with natal philopatry of hybrid offspring. Larger sample sizes and a modelling approach similar to that of Masello et al. (2019) could help distinguish these scenarios.

### Medium-billed *Pachyptila* species evolved independently through convergence

Bayesian trait reconstruction suggested an ancestral maternal bill width for *Pachyptila* of around 14 mm, which is roughly at a transition between extant narrow (11 mm) and medium-billed (14.2–18 mm) species (Fig. 2, Supplemental Material, Table S2). However, considering its wide range (Q1 = 11.9, Q3 = 16.4; Fig. 5), this ancestral bill width (14 mm) is still narrower than that of extant *P. macgillivrayi* (18 mm) and *P. salvini* (16.7 mm; Fig. 2, Supplemental Material, Table S2). Further ancestral trait reconstruction including the outgroup taxon suggests that bill width was even narrower further back in time, strongly implying that extant *Pachyptila* were derived from a thinner-billed ancestor, with medium bills > 16 mm and broad bills > 21 mm being derived character states. This is consistent with the evolution of specialised filtering lamellae along the sides of the upper mandible among the broader-billed *Pachyptila* taxa as a derived trait. However, we caution that our ancestral state reconstruction was based on a posterior distribution of mtDNA data, representing only maternal evolutionary events. A species tree reconstructed from autosomal loci would better account for introgression and so may be different to the mtDNA tree presented here. Whole genome sequencing of all species within the genus, with the aim of reconstructing a reliable species tree is thus a research priority.

Both mtDNA and nuclear markers revealed an evolutionary history for *P. macgillivrayi* that is distinct from that of *P. salvini*, despite the two species possessing similar bill phenotypes. Maternally, *P. macgillivrayi* is sister to *P. vittata*, whereas *P. salvini* is sister to *P. desolata* (Fig. 3). Microsatellite variation partitions *P. macgillivrayi* and Gough medium-billed as distinct from other species at *K* = 3, and with very little incoming gene flow, unlike the multilocus profile of *P. salvini*, where higher admixture can be observed (Fig. 4). Harper (1980) was thus correct in placing *P. macgillivrayi* as closer to *P. vittata* rather than *P. salvini* (Roux et al. 1986). Coalescent simulations of microsatellite DNA showed that *P. salvini*’s medium bill width (mean: this study, 16.7 mm, Fig. 2, 17.1 mm in Masello et al. (2019)) evolved through hybridisation of narrower- and broader-billed species, presumably conferring a feeding advantage as its intermediate bill width allowed it to feed on a wider variety of prey species than either of its parent species (Masello et al. 2019). Thus unlike *P. salvini*, which has a hybrid origin, our results show that *P. macgillivrayi* evolved after divergence from thinner-billed species (Fig. 3). Given this evolutionary scenario, it is likely that the broad bill of *P. vittata* was derived from a medium-billed ancestor, possibly similar in bill width to *P. macgillivrayi*. Therefore, the positions of similarly medium-billed *P. salvini* and *P. macgillivrayi* in different clades of the *Pachyptila* phylogeny strongly suggest mutually exclusive evolutionary histories for these species and, thus, the independent evolution of their phenotypes. As with *P. salvini*, an intermediate bill width (18 mm; Fig. 2, Supplemental Material, Table S2) might allow *P. macgillivrayi* to feed on a similarly wide range of prey species, providing a selective driver for the convergent evolution of the medium-billed phenotype.

### Status and conservation *P. macgillivrayi*

The conservation status of *P. macgillivrayi* was only assessed for the IUCN Red List of Threatened Species in 2016, because prior to the findings of our study it was not recognised as a species by BirdLife International. The population that breeds in the Indian Ocean is small; about 1000 pairs that were initially confined (150 pairs) to La Roche Quille, a rock stack 150 m off the coast of St Paul Island (Tollu 1984; Jiguet et al. 2007; Barbraud et al. 2021). The species was once abundant (> 10^5^ pairs) on Amsterdam Island and St Paul, but was extirpated from both islands by introduced predators (Worthy and Jouventin 1999; Jiguet et al. 2007). A significant number of prions have recolonised St Paul from La Roche Quille following the eradication of Black Rats *Rattus rattus* from St Paul in 1997 and European Rabbits *Oryctolagus cuniculus* in 1999 (Micol and Jouventin 2002; Griffiths 2011; Barbraud et al. 2021). Unfortunately, house mice *Mus musculus* remain on St Paul Island (Micol and Jouventin 2002), and predation by mice may slow or even halt the recovery of prions (Dilley et al. 2015). The population also is at risk from vagrant falcons that occasionally reach the island (Jiguet et al. 2007; Barbraud et al. 2021).

The population size of *P. macgillivrayi* on Gough Island is not well known, but based on their proportions in skua prey remains, they comprise at least 20% of prions breeding on the island (Jones 2018). Cuthbert (2004) estimated there were 1.75 million pairs of prions breeding on Gough Island in 2000. This estimate might be somewhat inflated, and numbers of prions probably are decreasing due to heavy predation on their eggs and chicks by introduced house mice (Cuthbert et al. 2013; Dilley et al. 2015), but it is still likely that Gough supports at least 10^5^ pairs of *P. macgillivrayi*; Caravaggi et al. (2019) estimated 875,000 pairs. Gough Island is thus home to more than 99% of the species’ global population*.* MacGillivray’s prion qualifies as Endangered under the IUCN Red List of Threatened Species Criterion B2a (two populations with a total breeding area < 70 km^2^). The species has experienced very poor breeding success (0–15%) in recent years due to mouse predation on Gough Island (Dilley et al. 2015), so its population is almost certainly decreasing. If so, it also qualifies as Endangered under Criterion B2b. The recognition of yet another globally threatened species that is virtually confined to Gough Island provides further impetus for the need to eradicate mice from the island (Jones et al. 2021).

## Conclusions

Our results suggest a different evolutionary history for *P. macgillivrayi* and *P. salvini*. We show that the intermediate bill width of *P. macgillivrayi* is not the product of interspecies introgression or hybrid speciation, but evolved through divergence. Remarkably, the newly described Gough Island population of medium-billed prions belong to the same evolutionary lineage as *P. macgillivrayi*, representing a new population of MacGillivray’s prion that originated through a colonisation event from St Paul Island. Unidirectional gene flow from Gough *P. vittata* into the Gough medium-billed population exists and merits future investigation. Given that the relict *P. macgillivrayi* population in the Indian Ocean is very small, our results demonstrating that the newly discovered medium-billed prions population on Gough corresponds to a new population of MacGillivray’s prion are of utmost relevance for the conservation of this species. Our results provide further evidence for the need to eradicate introduced house mice from Gough Island (Holmes et al. 2019; Jones et al. 2021).

## Supplementary Information

Below is the link to the electronic supplementary material.

Supplementary file1 (DOCX 6191 KB)

## Data Availability

DNA sequences: GenBank accession numbers are provided in Table 1. All data are available in the manuscript or in the Supplementary information file.
