# Supplementary material for: Independent evolution of intermediate bill widths in a seabird clade
Source: Mol Genet Genomics. 2021 Dec 18;297(1):183–98. doi: 10.1007/s00438-021-01845-3 (PMC8803701; doi:10.1007/s00438-021-01845-3)
Supplement: Supplementary file 1 — Supplementary file1 (DOCX 6191 KB) [file 438_2021_1845_MOESM1_ESM.docx]

**Supplementary information**

Independent evolution of intermediate bill widths in a seabird clade

Juan F. Masello, Peter G. Ryan, Lara D. Shepherd, Petra Quillfeldt, Yves Cherel, Alan J.D. Tennyson, Rachael Alderman, Luciano Calderón, Theresa L. Cole, Richard J. Cuthbert, Ben J. Dilley, Melanie Massaro, Colin M. Miskelly, Joan Navarro, Richard A. Phillips, Henri Weimerskirch, Yoshan Moodley

**Fig. S1** Genetic ancestry of all *Pachyptila* (Aves: Procellariiformes) breeding colonies sampled in this study as estimated by the program STRUCTURE from 18 microsatellite loci data set, and using the admixed model with correlated allele frequencies. Populations are coded with numbers and the number of individual samples is provided in brackets: 1) *P. macgillivrayi* from Saint Paul (12), 2) medium-billed Gough (10), 3) *P. vittata* from Gough (52), 4) *P. vittata* from Nightingale I., Tristan da Cunha (36), 5) *P. vittata* from Rangatira, Chathams. (30), 6) *P. salvini* from Marion (18), 7) *P. desolata* from South Georgia (35), 8) *P. desolata* from Kerguelen (38), 9) *P. desolata* from Macquarie (7).


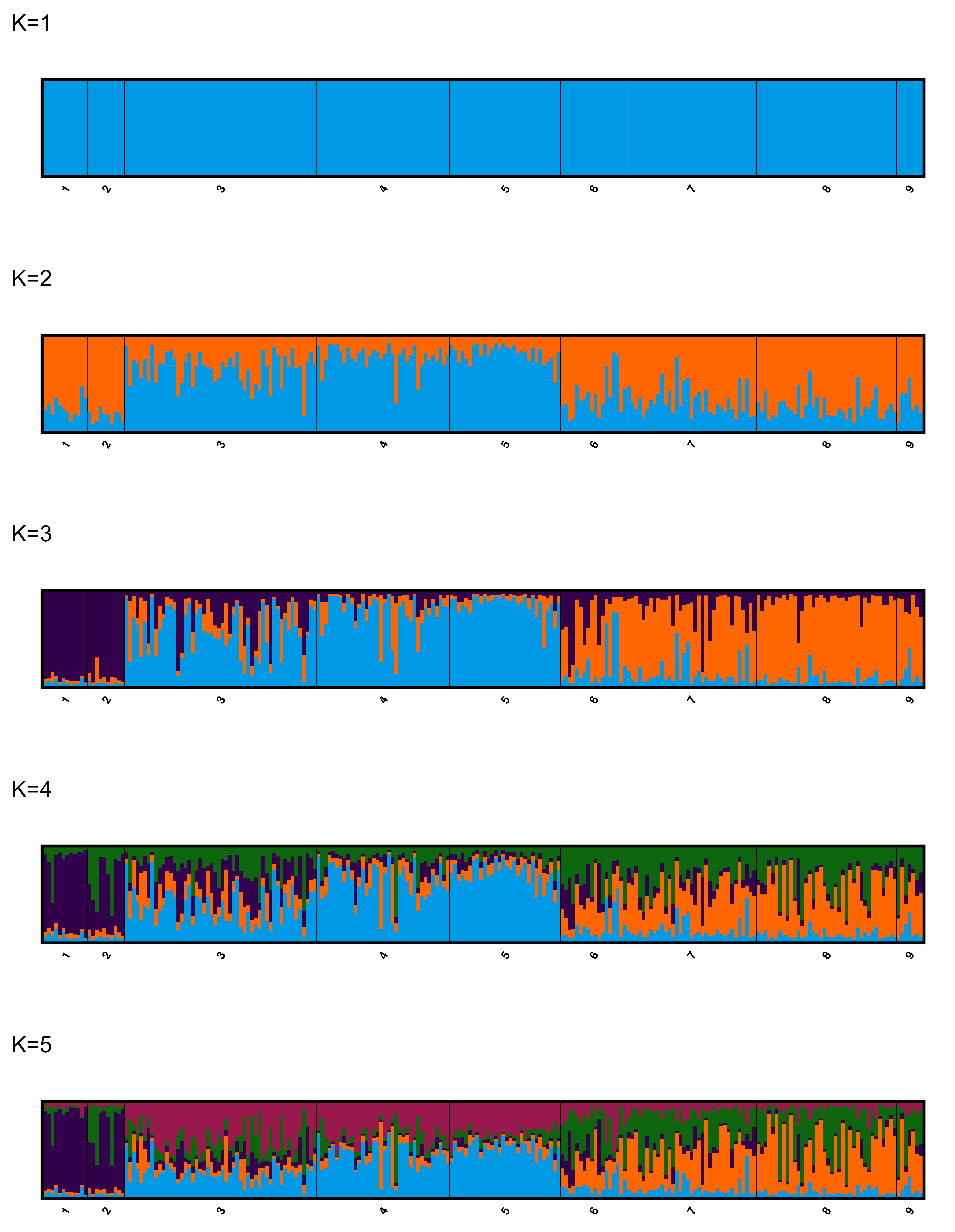


**Fig. S1** (cont.)


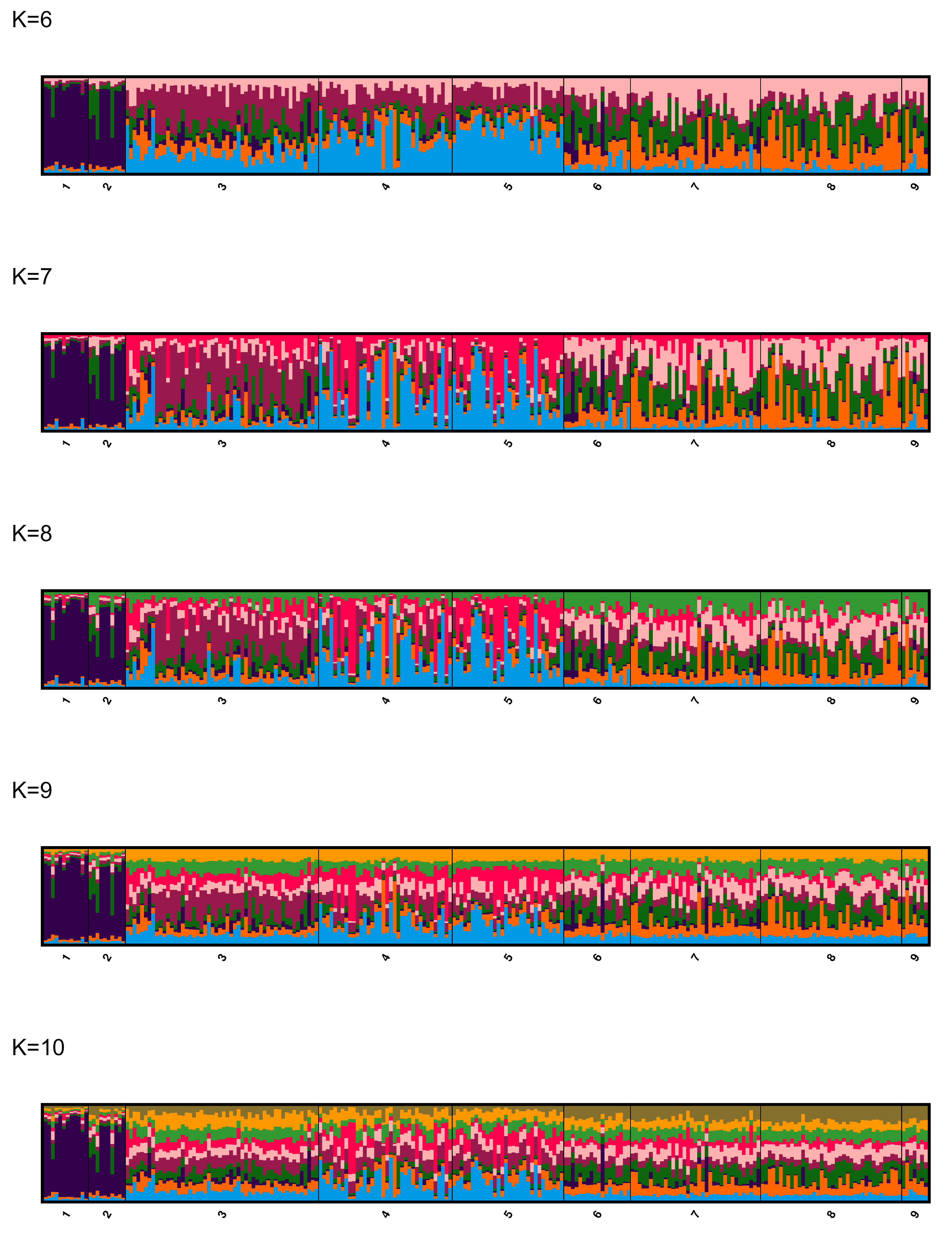


**Fig. S2** Bayesian ancestral state reconstruction of bill width at the root of the *Pachyptila* tree including the outgroup taxon *Halobaena caerulea*. Ingroup taxa include *P.* *desolata*, *P.* *salvini*, Gough medium-billed, *P.* *macgillivrayi*, *P.* *vittata*, *P.* *belcheri*, and *P.* *turtur.* The tree is displayed in Fig. 3B.

**

**

**Fig. S3** Variation in bill colour in broad-billed prions *Pachyptila vittata* (A–D) and Gough medium-billed prions *P. macgillivrayi* (E–F) breeding at Inaccessible Island, Tristan da Cunha (A, C) and at Gough Island (B, D–F). Photos by Peter Ryan, except the aberrant individual (D) by Karen Bourgeois.


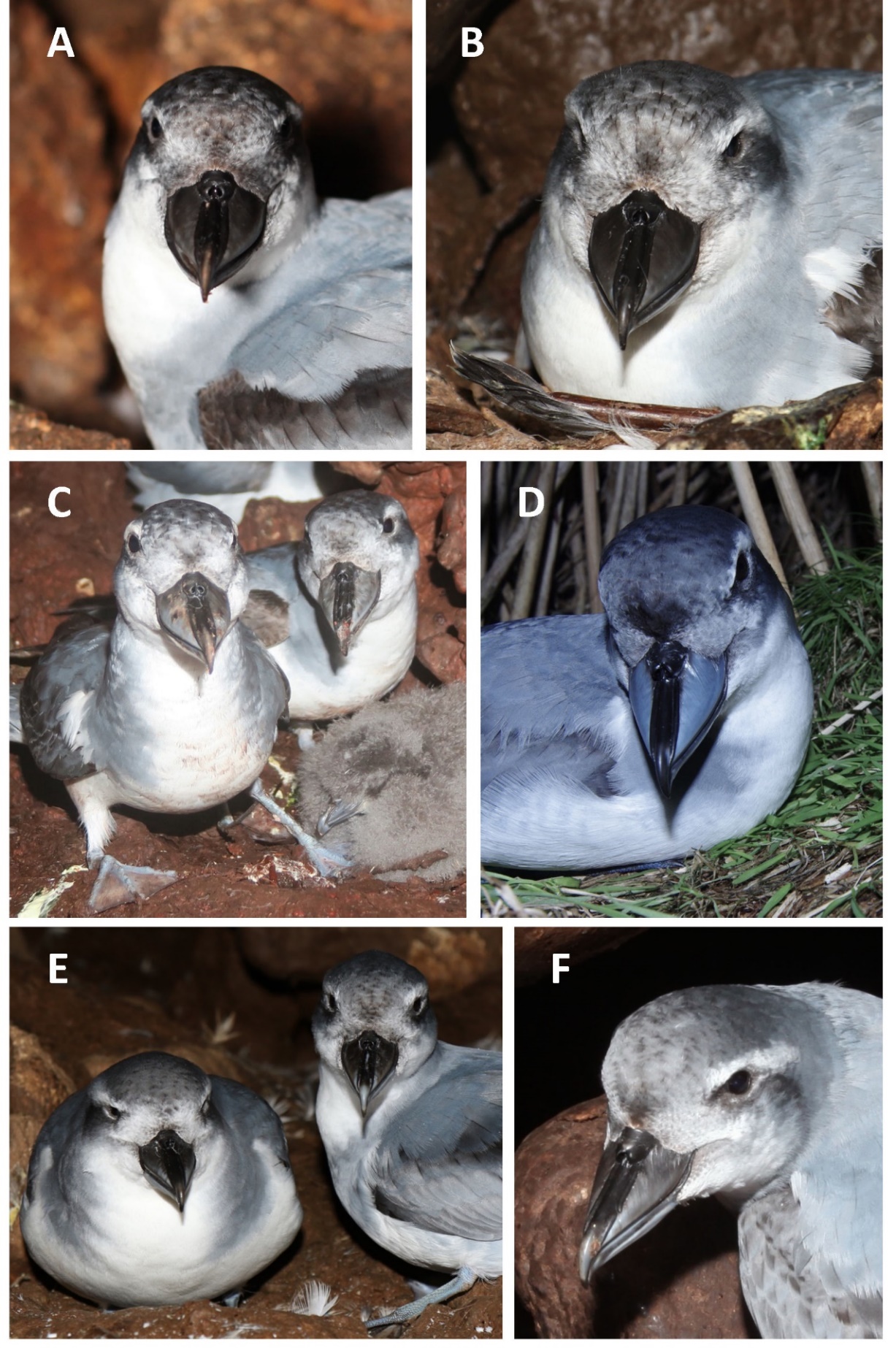


**Fig. S4** Variation in bill colour in broad-billed prions *Pachyptila vittata* breeding at Rangatira, Chatham Islands, showing two adults (A, C) and one fledgling (B). Photos by Petra Quillfeldt.


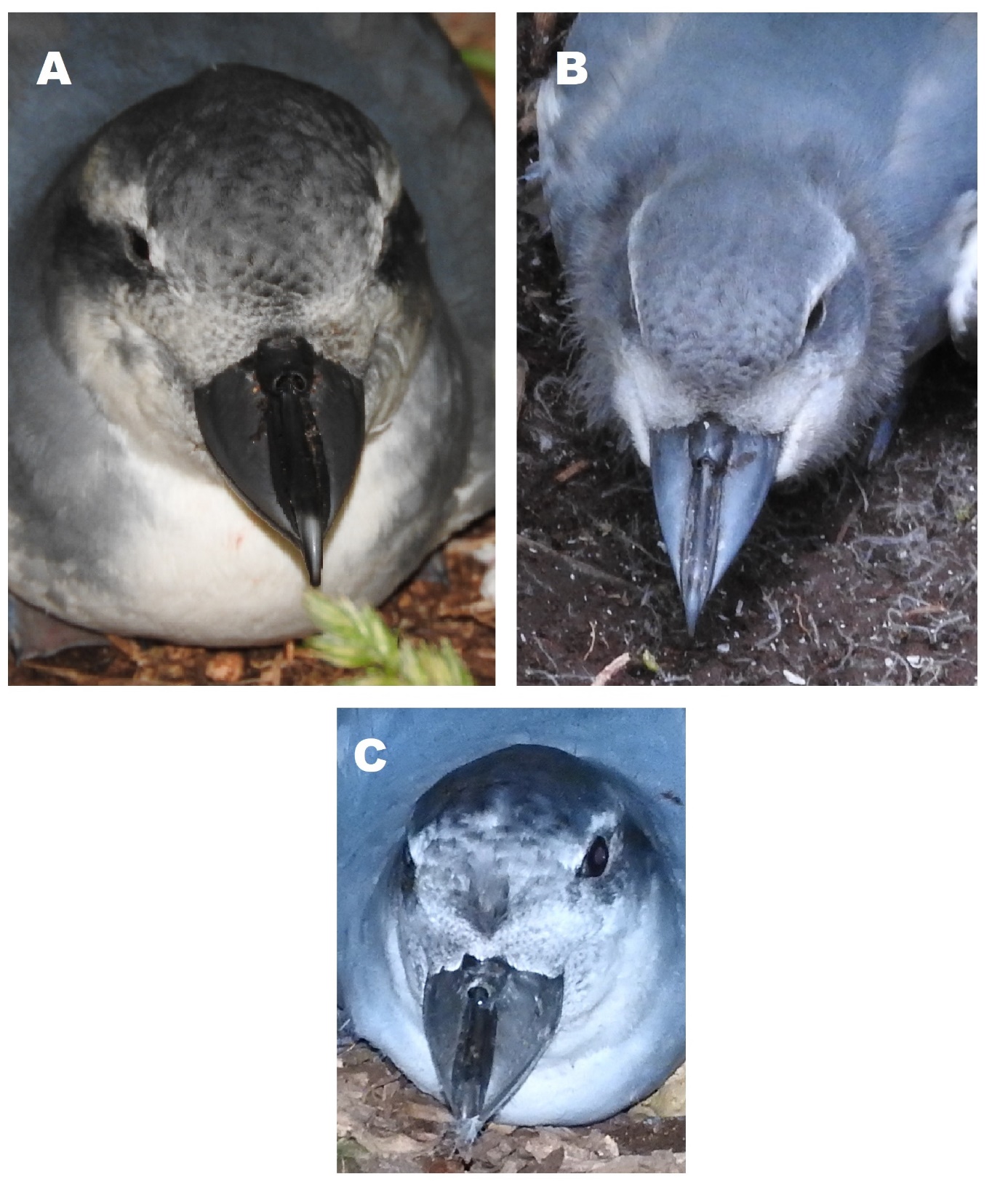


**Fig. S5** Variation in bill colour in putative Antarctic prions *Pachyptila desolata* at sea near Iles Crozet in December 2016: (A–B) unusual birds with blackish upper mandibles, (C–D) typical birds with pale blue-grey bill sides. Photos by Peter Ryan.

**Table S1** Subfossil bones of *Pachyptila macgillivrayi* from Amsterdam Island sampled for cytochrome *b* and cytochrome c oxidase subunit I (COI).

| NMNZ* registration number | GenBank accession number | |
| --- | --- | --- |
|  | cytochrome *b* | COI |
| S.34606.1 | no amplification | no amplification |
| S.34610.1 | no amplification | no amplification |
| S.34641.1 | no amplification | no amplification |
| S.34647.1 | no amplification | no amplification |
| S.34678.1 | no amplification | no amplification |
| S.34685.1 | no amplification | no amplification |
| S.34710.1 | MF159022 | KX092069 |
| S.34710.2 | no amplification | no amplification |
| S.34932 | no amplification | no amplification |
| S.35077.1 | no amplification | KX092070 |

*Museum of New Zealand Te Papa Tongarewa

**Table S2** Summary of bill width data from *Pachyptila* populations and *Halobaena caerulea* in this study.

| Taxon | *n* | Mean | S.D. | Min | Max | Median | 25% | 75% |
| --- | --- | --- | --- | --- | --- | --- | --- | --- |
| *H. caerulea* | 36 | 11.1 | 0.7 | 9.4 | 12.4 | 11.2 | 10.6 | 11.6 |
| *turtur* | 227 | 11.0 | 0.5 | 10 | 12.8 | 11 | 10.6 | 11.3 |
| *belcheri* | 168 | 11.0 | 0.3 | 9.9 | 12.5 | 10.8 | 10.8 | 11.3 |
| *desolata* | 64 | 14.2 | 1.0 | 12.2 | 16.5 | 14.0 | 13.5 | 14.9 |
| *salvini* | 43 | 16.7 | 0.9 | 15.2 | 18.6 | 16.6 | 16.1 | 17.2 |
| Gough medium-bill | 240 | 18.0 | 1.0 | 15.5 | 20.3 | 18 | 17.2 | 18.7 |
| *macgillivrayi* St Paul | 12 | 18.0 | 0.9 | 15.6 | 18.9 | 18.1 | 17.8 | 18.6 |
| *vittata* Gough | 424 | 21.8 | 0.8 | 19.2 | 24.4 | 21.8 | 21.2 | 22.3 |
| *vittata* Tristan | 120 | 21.4 | 1.0 | 19.1 | 24.2 | 21.5 | 20.8 | 22.0 |

*P. belcheri* and *P. turtur* data taken from Masello et al. (2019) and references therein.

**Table S3** Comparison of bill width data among the *Pachyptila* taxa measured in this study.

|  | *desolata* | Gough medium-bill | *macgillivrayi* St Paul | *salvini* | *vittata* Gough |
| --- | --- | --- | --- | --- | --- |
| Gough medium-bill | -5.224 |  |  |  |  |
| *P* | 0.000* |  |  |  |  |
| *macgillivrayi* St Paul | -2.384 | -0.051 |  |  |  |
| *P* | 0.009* | 0.480 |  |  |  |
| *salvini* | -1.769 | 2.331 | 1.228 |  |  |
| *P* | 0.039 | 0.010* | 0.110 |  |  |
| *vittata* Gough | -17.400 | -19.786 | -5.409 | -12.399 |  |
| *P* | 0.000* | 0.000* | 0.000* | 0.000* |  |
| *vittata* Tristan | -13.677 | -12.362 | -4.516 | -9.949 | 2.091 |
| *P* | 0.000* | 0.000* | 0.000* | 0.000* | 0.018* |

**Table S4** Posterior mean migration rates and standard deviation of the marginal posterior distribution for each estimate for all Pachyptila populations in this study.

| pop. 1 \ pop. 2 | *macgillivrayi* Saint Paul | medium-billed Gough | *vittata* Gough | *vittata* Tristan | *vittata* Chatham | *salvini* Marion | *desolata* South Georgia | *desolata* Kerguelen | *desolata* Macquarie |
| --- | --- | --- | --- | --- | --- | --- | --- | --- | --- |
| *macgillivrayi* Saint Paul | – | 0.0270  (0.0250) | 0.0312  (0.0285) | 0.0252  (0.0239) | 0.0243  (0.0228) | 0.0265  (0.0246) | 0.0255  (0.0236) | 0.0238  (0.0225) | 0.0260  (0.0242) |
| medium-billed Gough | **0.2815**  (0.0383) | – | 0.0766  (0.0476) | 0.0318  (0.0295) | 0.0306  (0.0281) | 0.0966  (0.0753) | 0.0408  (0.0423) | 0.0407  (0.0371) | 0.1464  (0.0789) |
| *vittata* Gough | 0.0070  (0.0068) | 0.0064  (0.0063) | – | 0.0071  (0.0069) | **0.3201**  (0.0125) | 0.0069  (0.0068) | 0.0096  (0.0086) | 0.0083  (0.0078) | 0.0064  (0.0061) |
| *vittata* Tristan | 0.0104  (0.0100) | 0.0108  (0.0108) | **0.3202**  (0.0128) | – | **0.3179**  (0.0147) | 0.0165  (0.0157) | 0.0158  (0.0157) | 0.0250  (0.0222) | 0.0483  (0.0276) |
| *vittata* Chatham | 0.0106  (0.0104) | 0.0107  (0.0104) | 0.0119  (0.0115) | 0.0275  (0.0207) | – | 0.0129  (0.0122) | 0.0208  (0.0169) | 0.0110  (0.0106) | 0.0112  (0.0108) |
| *salvini* Marion | 0.0443  (0.0358) | 0.0334  (0.0274) | **0.2846**  (0.0290) | **0.2695**  (0.0332) | 0.2167  (0.0465) | – | **0.3119**  (0.0201) | **0.3123**  (0.0200) | 0.2419  (0.0688) |
| *desolata* South Georgia | 0.0117  (0.0112) | 0.0115  (0.0109) | **0.2748**  (0.0331) | **0.3144**  (0.0160) | **0.2867**  (0.0404) | 0.0107  (0.0105) | – | 0.0149  (0.0136) | 0.0097  (0.0096) |
| *desolata* Kerguelen | 0.0149  (0.0127) | 0.0103  (0.0100) | 0.1919  (0.0516) | **0.3151**  (0.0158) | **0.2791**  (0.0310) | 0.0090  (0.0089) | **0.3249**  (0.0083) | – | 0.0096  (0.0092) |
| *desolata* Macquarie | 0.1195  (0.0537) | 0.0882  (0.0779) | **0.2904**  (0.0378) | 0.1470  (0.0636) | **0.2800**  (0.0475) | 0.2299  (0.0730) | **0.2923**  (0.0368) | **0.2912**  (0.0367) | – |

Mean migration rates (m) as a proportion from 0−1 and standard deviation (SD). Values in bold highlight exceptionally high migration rates. Values below the diagonal correspond to m[1][2] (± SD) which is the fraction of individuals in population 1 (column far left) that are migrants derived from population 2 (top line) per generation. Values above the diagonal correspond to m[2][1] (± SD).
